# Diagnosis and Supportive Management of Fucosidosis: A Case Report

**DOI:** 10.7759/cureus.6139

**Published:** 2019-11-12

**Authors:** Arpanjeet Kaur, Arshdeep S Dhaliwal, Hillary Raynes, Thomas P Naidich, David M Kaufman

**Affiliations:** 1 General Medicine, Sibia Healthcare Pvt. Ltd., Sangrur, IND; 2 General Medicine, Madhu Nursing Home, Patiala, IND; 3 Pediatric Neurology, Mount Sinai Hospital, New York, USA; 4 Radiology, Mount Sinai Hospital, New York, USA

**Keywords:** diagnosis, fucosidosis, fuca1, supportive management

## Abstract

Fucosidosis is a lysosomal storage disease, resulting from a deficiency of the enzyme alpha-L-fucosidase. We present the case of an affected female with numerous manifestations, clinically and radiographically. In fucosidosis, advanced interventions are not always necessary to have rewarding outcomes. In fact, early diagnosis and management of the symptoms with a multi-systemic supportive care approach can improve the quality of life and may also prolong the life of those patients diagnosed with fucosidosis.

## Introduction

Fucosidosis is a very rare, autosomal, recessive genetic disorder associated with a mutation in the FUCA1 gene, resulting in a severe deficiency of the enzyme alpha-L-fucosidase in lysosomes. A total of 100 cases of fucosidosis have been reported until now [1-2]. Deficiency of this enzyme leads to the accumulation of fucose-containing glycolipids and other substances in various organs, which display a wide array of symptoms, such as severe global developmental delay (95%), muscle stiffness (87%), coarse facies (79%), recurrent respiratory infections (78%), abnormal bone development (58%), tortuous conjunctival vessels (53%), clusters of enlarged blood vessels on the skin (52%), abnormal enlargement of visceral organs (44%), and seizures (38%) [3-6]. Studies have shown that around 60% of the patients die before the age of 10 years, secondary to recurrent pulmonary infections and neurological deterioration [7].

We present a 16-year-old female who was diagnosed with fucosidosis at two and a half years of age, with a majority of these systems involved. Various diagnostic tests like magnetic resonance imaging (MRI), magnetic resonance spectroscopy (MRS), and genetic testing helped us to reach a definitive diagnosis. Keeping the severity and extensive involvement of different systems in mind, we believe that our supportive management plan could be one of the reasons for her survival into her second decade of life.

## Case presentation

The patient is a 16-year-old Bulgarian girl, born prematurely at 25 weeks of gestation, followed by a postnatal newborn intensive care unit (NICU) stay. She met her developmental milestones at age-appropriate intervals consistent with corrections for gestational age. She had 15 words by 18 months, and she walked at two years of age (corrected age of 20 months). She began to lose language at 24 months of age and loss of motor milestones ensued after that. The regression that occurred in her third year of life brought concerns for a primary central nervous system disorder and, most likely, a metabolic disorder. The work-up was initiated by her neurologist and geneticist at the time with a high possibility of glycoprotein storage disorders like aspartylglucosaminuria, fucosidosis, galactosialidosis, alpha-mannosidosis, beta-mannosidosis, mucolipidosis type two, Schindler disease, and sialidosis.

She was diagnosed with fucosidosis at around two and a half years of age through enzyme analysis, and over the ensuing few years, there were numerous concerns for the continued regression, failure to thrive, multiple contractures, hip dysplasia, and telangiectatic vessels in her conjunctivae. Her mental functioning at five years of age was equivalent to someone less than 12 months old. Her symptoms worsened with time, and she stopped walking at around seven years of age. Scoliosis was diagnosed at seven to eight years of age, which contributed to recurrent pneumonia. She was hospitalized for five episodes of pneumonia between the ages of 12 and 13. At age 14, she experienced her first generalized tonic-clonic seizure and was subsequently diagnosed with epilepsy. Additionally, she developed dystonic movements of the tongue, face, neck, and right upper and lower extremities. She has coarse gargoyle features, protruding tongue, macroglossia, recalcitrant nasal polyposis, chronic respiratory distress with recurrent infections, and gastroesophageal reflux disease (GERD). She is globally impaired and is unable to speak, but her receptive language is intact by the parents’ report. She has angiokeratomas on her lower extremities and trunk. Her extremities are malformed due to the extensive dysostosis multiplex, which has caused significant scoliosis with malrotation of her chest cavity, multiple contractures, with foreshortened limbs. Osteopenia is present without fractures. Her height and weight percentiles for age are far below the third percentile curves.

Fucosidosis was not diagnosed at our institution but genetic testing, MRI and MR spectroscopy were concurrently done to confirm the diagnosis. On genetic testing, nucleotide c. 194G>A (p.Gly65sp) change is seen on exon 1 with heterozygosity in our patient. This change has been classified as ‘likely’ pathogenic. Both parents were also noted to be carriers of her mutation in the FUCA1 gene, but her elder sister has no manifestations of this disease.

**Figure 1:**
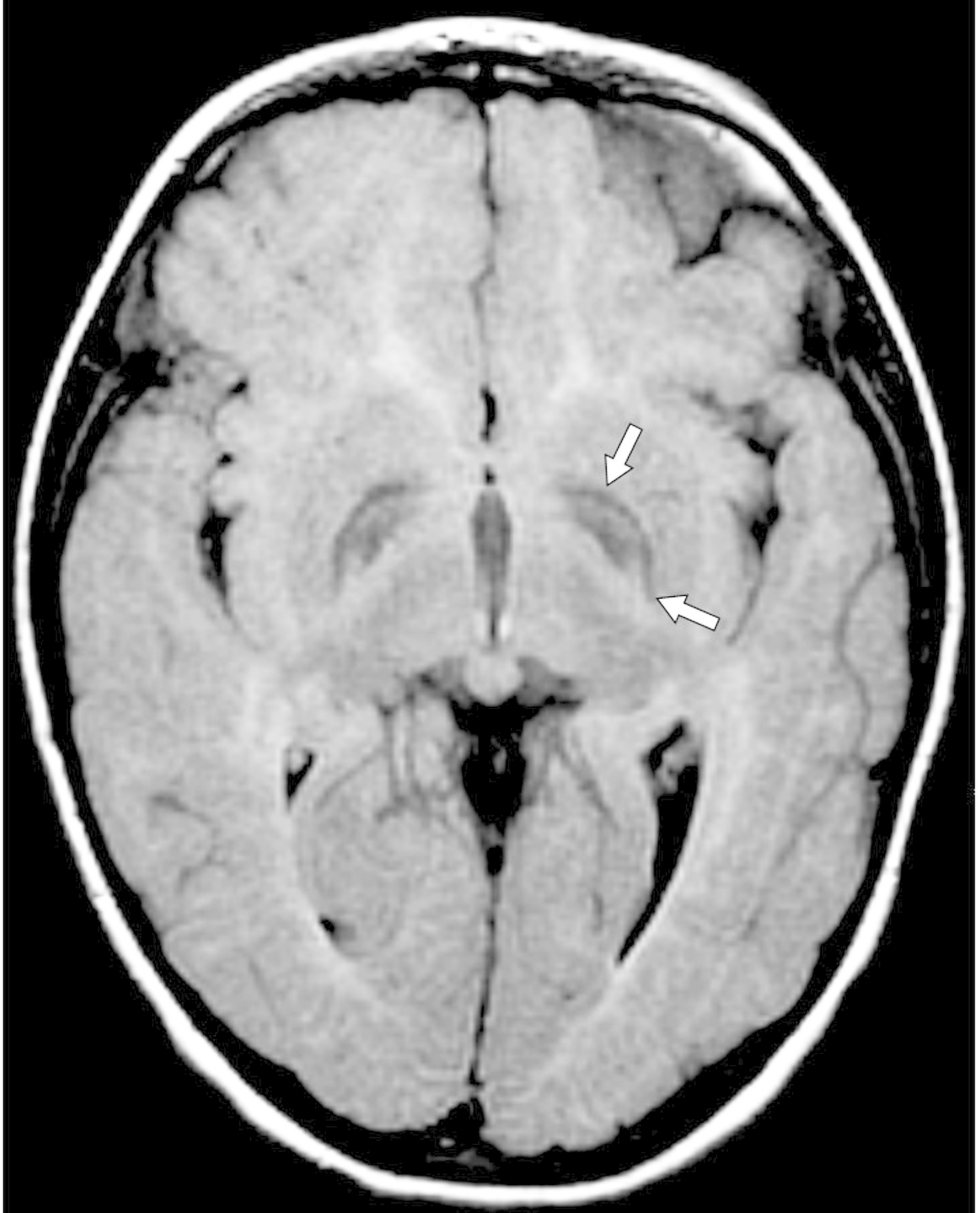
Globi pallidi showing symmetrical high T1 and low T2/ T2-fluid-attenuated inversion recovery (FLAIR) signal with characteristic curvilinear bands (horizontal plane)

**Figure 2:**
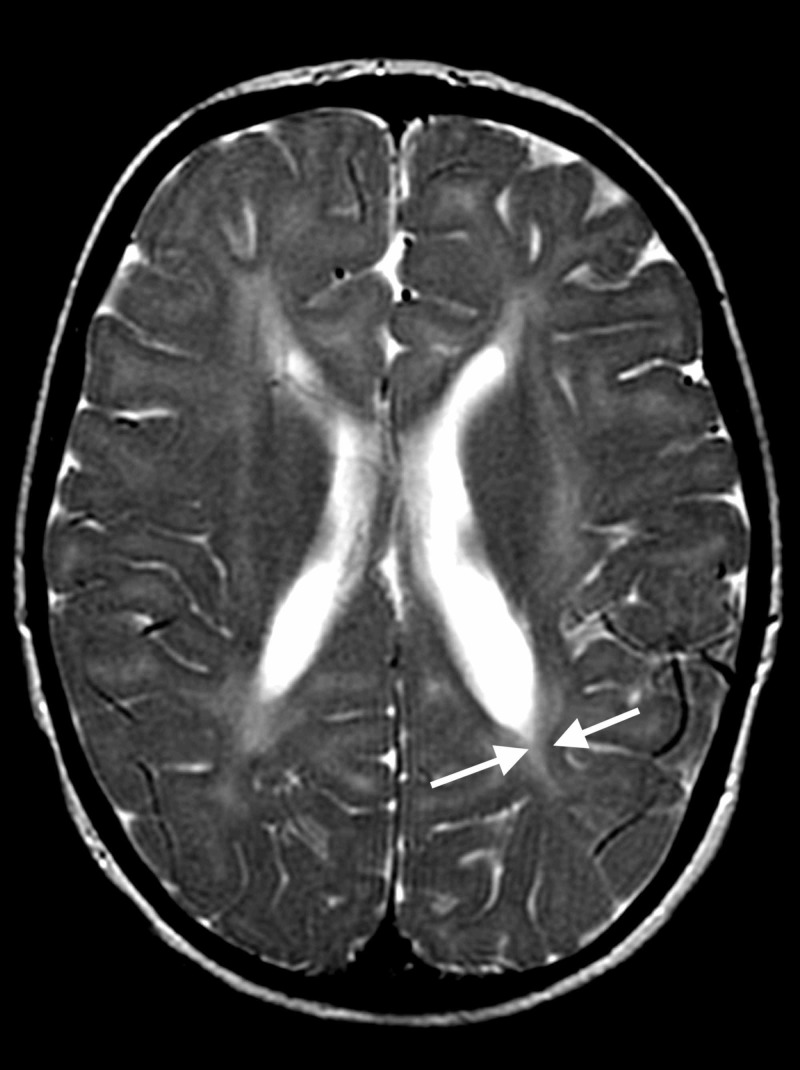
Atrophic enlargement of the lateral ventricles with reduced white matter volume, symmetrical increase in T2 signal intensity in subcortical and deep white matter (horizontal plane)

**Figure 3:**
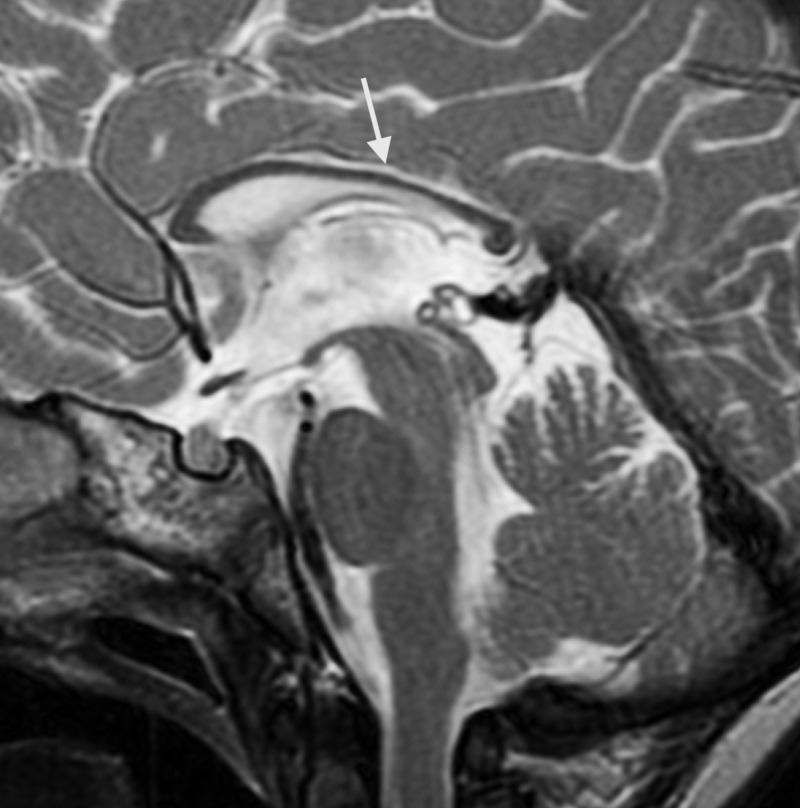
Thin corpus callosum (sagittal plane)

**Figure 4:**
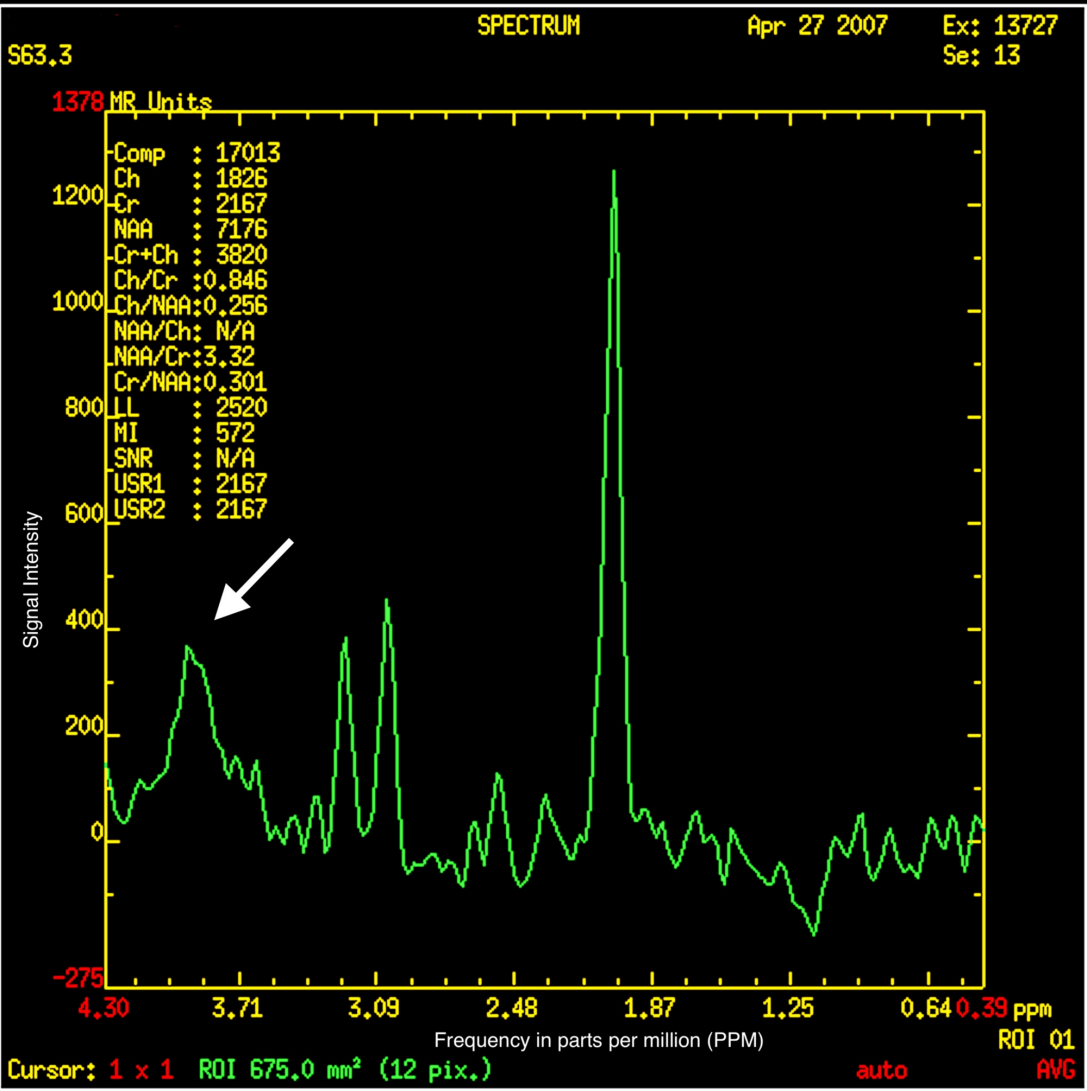
Magnetic resonance spectroscopy showing a prominent peak at 3.8-3.9 parts per million (PPM)

**Figure 5:**
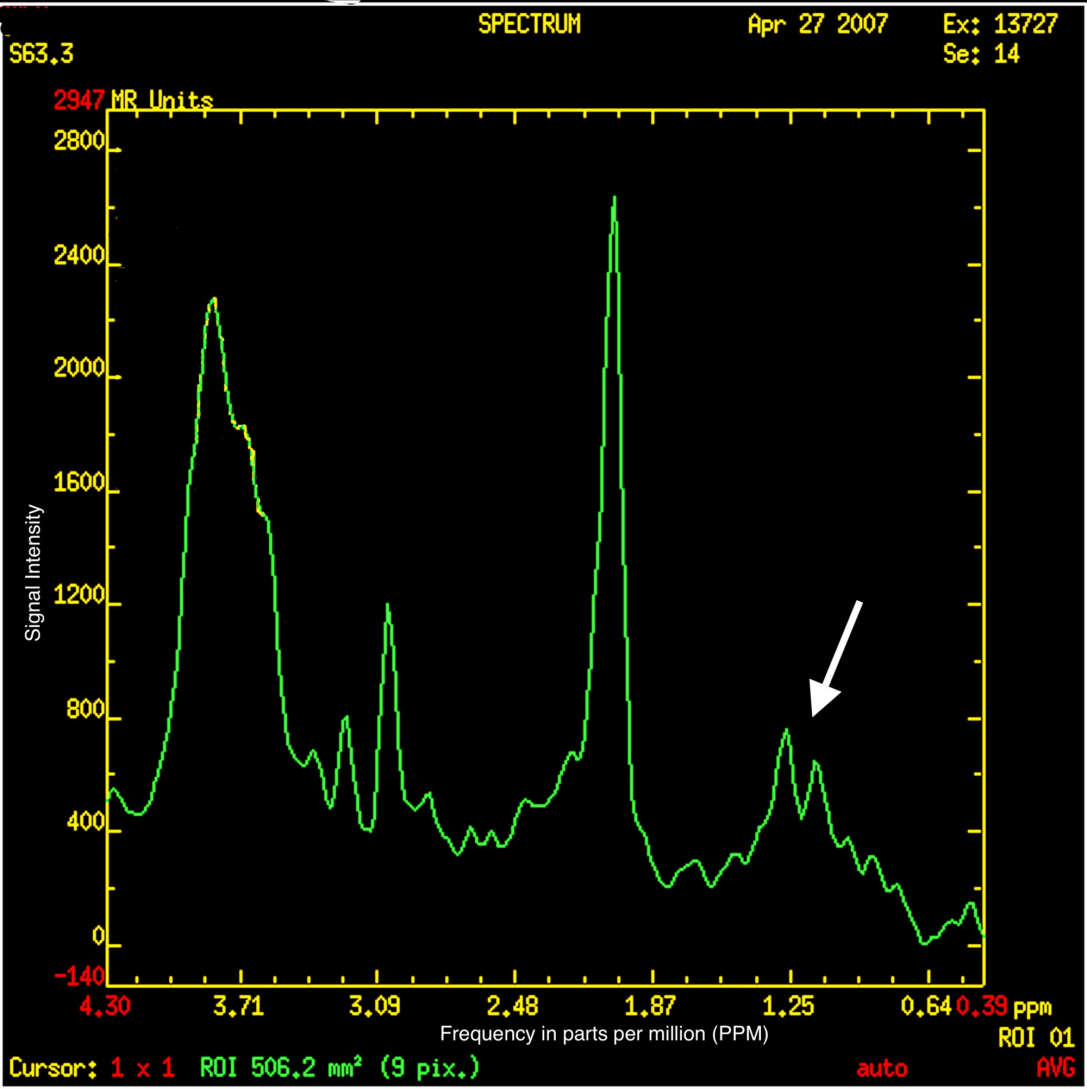
Magnetic resonance spectroscopy showing a doublet peak at 1.2 parts per million (PPM)

On MRI, done at four years of age, the globi pallidi show symmetrical high T1 and low T2/T2-fluid-attenuated inversion recovery (FLAIR) signal with characteristic curvilinear bands of increased T2 signal intensity along (i) the lateral medullary lamina between the putamen and the external nucleus of globus pallidus (GPe) and (ii) the internal medullary lamina between GPe and the internal nucleus (GPi) of the globus pallidus (Figure 1) [8-10]. The subcortical and deep white matter show a variably severe, symmetrical increase in T2 signal intensity and reduced white matter volume with corresponding atrophic enlargement of the lateral ventricles (Figure 2). The corpus callosum is thin (Figure 3). The superior vermis may be atrophic. MR spectroscopy shows a prominent peak at 3.8-3.9 ppm (Figure 4) and a doublet peak at 1.2 ppm (Figure 5).

Following the confirmation of her diagnosis with genetic testing, enzyme analysis, and neuroimaging, bone marrow transplant (BMT), hematopoietic stem cell transplantation (HSCT), and umbilical cord blood transplant (UCBT) were recommended to the family as possible treatments. However, due to her irreversible advanced disease burden, the family opted not to pursue any transplant and decided to treat symptomatically.

Since her diagnosis, a multi-systemic care approach was designed in coordination with multiple subspecialties to establish a management plan, which would prioritize the most vital symptoms for her comfort and long-term survival. As respiratory (both upper and lower) and neurological symptoms are the most likely cause of death in patients with fucosidosis before 10 years of age, it was imperative for her caregivers to address these issues early on. Respiratory-wise, upper respiratory tract symptoms involved a heavy burden of nasal polyps managed with nasal polyp removal, bimonthly nasal endoscopy, and daily saline sinus rinse mixed with gentamicin and budesonide. The lower respiratory tract symptoms were managed with oxygen, albuterol, and fluticasone inhalers, nebulized beclomethasone, oral clindamycin, and an airway clearance system. The airway clearance system (the vest) is employed as a mechanical pulmonary toilet on a daily basis to dislodge and mobilize mucus to the most proximal airways, preventing atelectasis and pneumonia. Neurologically, seizures were controlled with anti-epileptic therapy (levetiracetam) and dystonia was treated with baclofen, botulinum toxin injections, and physical, occupational, and massage therapy. Other supportive care management, as described in Table 1, played an important role in managing her symptoms. Her stable condition at the age of 16 is a reflection of the efficacy that the dedicated and compulsory care directed at each of her affected systems has helped to improve her quality of life and allow for comfort despite a heavy disease burden.

**Table 1:** Associated symptoms along with the pertinent management plan †: GERD: Gastroesophageal reflux disease ENT: Ear, nose, throat

SYMPTOMS	MANAGEMENT
Spasticity and dystonia	Baclofen, botulinum toxin injections, physical, occupational and massage therapy
Seizures	Anti-epileptic therapy
Respiratory	Oxygen through a nasal cannula, albuterol, fluticasone, nebulized beclomethasone, oral clindamycin, and the airway clearance system (the vest); oral suction as needed for excessive secretions
Gastrointestinal	Gastrostomy tube, feeding therapy, famotidine (GERD)†
ENT: recalcitrant nasal polyposis	Nasal polyp removal, bimonthly nasal endoscopy, daily saline sinus rinse mixed with gentamicin and budesonide
Diet	Pureed food
Speech	Speech and language therapy

## Discussion

Fucosidosis is a rare, inherited, autosomal recessive lysosomal storage disorder with an estimated frequency of below one in 200,000 live births [11]. It is sub-classified into two types. Type one is the more severe form, beginning by six months and leading to rapid neurologic deterioration and demise by the first decade of life [8]. Type two has a relatively moderate course compared to type one with a slower neurologic deterioration that begins at around 18 months to two years of age [1]. This classification is under debate since some studies suggest that the two types are actually a single disorder with signs and symptoms ranging only in severity. Life expectancy depends on the type and severity of the disease, with high mortality rates less than 10 years of age [6-7,12-13].

The survival of our patient to age 16 despite her severe disease burden confirms for us that timely and aggressive supportive and conservative care can sustain the systems involved to provide the best quality of life possible in the absence of targeted treatment, especially when the disease has progressed beyond optimal interventions like bone marrow transplant (BMT), hematopoietic stem cell transplantation (HSCT), and umbilical cord blood transplant (UCBT).

The clinical features of fucosidosis include severe intellectual disability, progressive spastic quadriplegia, coarse facies, growth retardation, visceromegaly, angiokeratoma corporis diffusum, recalcitrant nasal polyposis, recurrent bronchopneumonias, seizures, and variable degrees of dysostosis multiplex [7,12-14]. Studies have shown that the majority of those affected develop severe progressive spastic quadriplegia rather than dystonia. The involvement of the globus pallidus on MRI, as witnessed in our case, further strengthens the potential for dystonic movements in fucosidosis, as has been rarely reported (Figure 1) [12,15-16].

Diagnosing fucosidosis can be very challenging, as advanced radiologic techniques and advanced genetic testing is very limited in certain parts of the world, especially in developing nations. Although genetic testing is the best way to determine inherited diseases, the pattern of MRI and MRS findings, along with clinical signs and symptoms, can help physicians diagnose fucosidosis. On MRI, fucosidosis may be seen to affect both the gray matter and the white matter of the brain, with variable severity in different patients at differing ages. The low T2 signal within the globus pallidus may reflect the accumulation of fucose-rich oligosaccharides and glycolipids, as suggested by pathology reports of neutral fats, cholesterol esters, and macrophages laden with triglycerides in neuropathological specimens. The increased T1 signal intensity remains enigmatic. The increased T2 signal within the white matter has been shown to represent incomplete myelination in some cases but myelin loss with gliosis in others [17]. The white matter changes have been attributed to reversible hypomyelination in some cases and to white matter loss with gliosis in others [1,10,13]. On MR spectroscopy, the peak at 3.8-3.9 ppm has been attributed to accumulating carbohydrate-containing macromolecules. A doublet peak at 1.2 ppm that inverts at TE 135 suggests fucose [17].

Managing fucosidosis can be challenging, as “one size does not fit all.” Studies have shown that various management techniques like bone marrow transplant (BMT), hematopoietic stem cell transplantation (HSCT), and umbilical cord blood transplantation (UCBT) may lead to positive outcomes, but long-term results and survival from these interventions have not yet been determined [1,10,16]. As seen in our case, care directed to support multi-organ involvement and timely management of the associated complications may improve the quality of life and prolong life expectancy in such patients.

## Conclusions

This case of severely symptomatic fucosidosis exemplifies the wide spectrum and variability in the disease burden. Fucosidosis should be suspected in cases with clinical features along with the findings described on MRI, MRS, and genetic testing. This case illustrates that adopting a multi-systemic care plan (referenced in Table 1) is a viable option that may improve the quality of life and provide for supportive and maximal care for those who may not choose or may not be eligible for transplantation therapies.

## References

[1] Jiang M, Liu S, Jiang H (2017). Brain abnormalities in fucosidosis: transplantation or supportive therapy. Metab Brain Dis.

[2] Turner B, Beratis N, Turner V, Hirschhorn KJN (1975). Silent allele as genetic basis of fucosidosis. Nature.

[3] Willems PJ, Gatti R, Darby JK, Romeo G, Durand P, Dumon JE, O'Brien JS (1991). Fucosidosis revisited: a review of 77 patients. Am J Med Genet.

[4] Willems PJ, Seo H-C, Coucke P, Tonlorenzi R, O'Brien JS (1999). Spectrum of mutations in fucosidosis. Eur J Med Genet.

[5] Sánchez LR, Oatts JT, Duncan JL, Packman S, Moore AT (2016). Ocular findings in a patient with fucosidosis. Am J Ophthalmol Case Rep.

[6] Bharati A, Higgins C, Ellis I, Wraith J (2007). Fucosidosis: a therapeutic challenge. Pediatr Dermatol.

[7] Turkia HB, Tebib N, Azzouz H (2008). Phenotypic spectrum of fucosidosis in Tunisia. J Inherit Metab Dis.

[8] Galluzzi P, Rufa A, Balestri P, Cerase A, Federico A (2001). MR brain imaging of fucosidosis type I. AJNR Am J Neuroradiol.

[9] Provenzale JM, Barboriak DP, Sims K (1995). Neuroradiologic findings in fucosidosis, a rare lysosomal storage disease. AJNR Am J Neuroradiol.

[10] Miano M, Lanino E, Gatti R (2001). Four year follow-up of a case of fucosidosis treated with unrelated donor bone marrow transplantation. Bone Marrow Transplant.

[11] Panmontha W, Amarinthnukrowh P, Damrongphol P, Desudchit T, Suphapeetiporn K (2016). Novel mutations in the FUCA1 gene that cause fucosidosis. Genet Mol Res.

[12] Wali G, Wali G, Sue CM, Kumar KR (2019). A novel homozygous mutation in the FUCA1 gene highlighting fucosidosis as a cause of dystonia. Case report and literature review. Neuropediatrics.

[13] Zubarioglu T, Kiykim E, Zeybek CA, Cansever MS, Benbir G, Aydin A, Yalcinkaya C (2015). Clinical and neuroradiological approach to fucosidosis in a child with atypical presentation. Ann Indian Acad Neurol.

[14] Peng T, Modi VK, Pearlman AN (2017). Recalcitrant chronic rhinosinusitis in the setting of fucosidosis, a rare lysosomal storage disorder. Int J Pediatr Otorhinolaryngol.

[15] Gautschi M, Merlini L, Calza A-M, Hayflick S, Nuoffer J-M, Fluss J (2014). Late diagnosis of fucosidosis in a child with progressive fixed dystonia, bilateral pallidal lesions and red spots on the skin. Eur J Paediatr Neurol.

[16] Gordon B, Gordon K, Seo H, Yang M, DiCioccio R, O'Brien JS (1995). Fucosidosis with dystonia. Neuropediatrics.

[17] Ediz SS, Aralasmak A, Yilmaz TF, Toprak H, Yesil G, Alkan A (2016). MRI and MRS findings in fucosidosis; a rare lysosomal storage disease. Brain Dev.

